# Supplementary material for: Causal Relationship between Aging and Anorexia Nervosa: A White-Matter-Microstructure-Mediated Mendelian Randomization Analysis
Source: Biomedicines. 2024 Aug 16;12(8):1874. doi: 10.3390/biomedicines12081874 (PMC11351342; doi:10.3390/biomedicines12081874)
Supplement: Supplementary file 1 [file biomedicines-12-01874-s001.zip › Supplementary Figure/Figure S8/Leaveoneout plot of MR analysis for TL to ubm-b-1456.pdf]

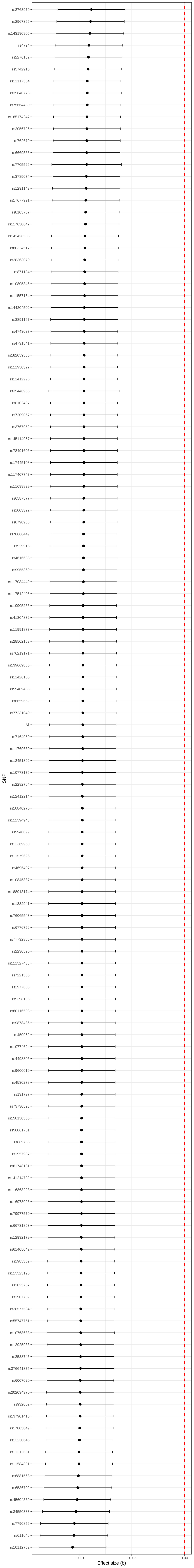
rs2763979
rs2967355
rs143190905
rs4724
rs2276182
rs5742915
rs11117354
rs35640778
rs75664430
rs185174247
rs2056726
rs762679
rs6669563
rs7705526
rs3785074
rs1291143
rs17677991
rs8105767
rs117630647
rs142426306
rs80324517
rs28363070
rs871134
rs10805346
rs11557154
rs144204502
rs3891167
rs4743037
rs4731541
rs182059586
rs111950327
rs11412296
rs35446936
rs8102497
rs7209057
rs3767952
rs145114957
rs78491606
rs17445108
rs117407747
rs11699829
rs6587577
rs1003322
rs6790988
rs76666449
rs939916
rs4616688
rs9955360
rs117034449
rs117512405
rs10905255
rs41304832
rs11991877
rs28502153
rs76219171
rs139669835
rs11426156
rs59409453
rs6659669
rs77231040
All
rs7164950
rs11769630
rs12451892
rs10773176
rs2282764
rs12412214
rs10840270
rs112394943
rs9940099
rs12369950
rs11579626
rs4695407
rs10845387
rs188918174
rs1332941
rs76065543
rs6776756
rs77732866
rs2230590
rs111527438
rs7221585
rs2977608
rs9398196
rs80116508
rs9878436
rs450962
rs10774624
rs4498805
rs9600019
rs4530278
rs131797
rs73730598
rs150150565
rs56061761
rs869785
rs1957937
rs61748181
rs141214782
rs116863223
rs16978028
rs79977579
rs66731853
rs12932179
rs61405042
rs1985369
rs113525195
rs1023767
rs1907702
rs28577594
rs55747751
rs10768683
rs12925933
rs2538745
rs376641875
rs6007020
rs202034370
rs932002
rs137901416
rs17803849
rs13230646
rs11212631
rs11584821
rs6881568
rs6536702
rs45604339
rs34550383
rs7790856
rs611646
rs10112752
SNP
−0.10
−0.05
0.00
Effect size (b)
